# Photoacoustic Tomography with a Ring Ultrasound Transducer: A Comparison of Different Illumination Strategies

**DOI:** 10.3390/app9153094

**Published:** 2019-07-31

**Authors:** Naser Alijabbari, Suhail S. Alshahrani, Alexander Pattyn, Mohammad Mehrmohammadi

**Affiliations:** 1 Department of Biomedical Engineering, Wayne State University, Detroit, MI 48201, USA; 2 Department of Electrical and Computer Engineering, Wayne State University, Detroit, MI 48201, USA; 3 Barbara Ann Karmanos Cancer Institute, Detroit, MI 48201, USA

**Keywords:** full-ring illumination, diffused-beam illumination, point source illumination, ultrasound tomography (UST), photoacoustic tomography (PAT)

## Abstract

Photoacoustic (PA) imaging is a methodology that uses the absorption of short laser pulses by endogenous or exogenous chromophores within human tissue, and the subsequent generation of acoustic waves acquired by an ultrasound (US) transducer, to form an image that can provide functional and molecular information. Amongst the various types of PA imaging, PA tomography (PAT) has been proposed for imaging pathologies such as breast cancer. However, the main challenge for PAT imaging is the deliverance of sufficient light energy horizontally through an imaging cross-section as well as vertically. In this study, three different illumination methods are compared for a full-ring ultrasound (US) PAT system. The three distinct illumination setups are full-ring, diffused-beam, and point source illumination. The full-ring system utilizes a cone mirror and parabolic reflector to create the ringed-shaped beam for PAT, while the diffuse scheme uses a light diffuser to expand the beam, which illuminates tissue-mimicking phantoms. The results indicate that the full-ring illumination is capable of providing a more uniform fluence irrespective of the vertical depth of the imaged cross-section, while the point source and diffused illumination methods provide a higher fluence at regions closer to the point of entry, which diminishes with depth. In addition, a set of experiments was conducted to determine the optimum position of ring-illumination with respect to the position of the acoustic detectors to achieve the highest signal-to-noise ratio.

## 1. Introduction

Breast cancer is a significant health problem not only in the United States but globally and was the second leading cause of cancer-related death in the United States in 2018 [1]. Mammography, MRI (Magnetic Resonance Imaging), and B-mode ultrasound are the three most common imaging modalities used for breast cancer screening [2,3]. However, each of these modalities has its own unique shortcomings. The sensitivity of mammography in detecting breast lesions decreases in women with high-density breast tissue, and high-density breasts are considered to be more at risk for developing breast cancer [3,4]. In dense breasts, MRI can be used in conjunction with breast mammography to detect breast tumors [5,6]. Nevertheless, the operational cost and availability of MRI imaging limit the accessibility of this modality. Conventional B-mode ultrasound (US) is a high-sensitivity, non-ionizing, and low-cost tool that is widely used for screening various types of human tissues [7,8]. However, false positives due to ultrasound screening result in many unnecessary biopsies [9,10]. Therefore, a more effective breast cancer screening tool is sought.

Photoacoustic tomography (PAT) is an imaging methodology that uses light absorption by endogenous or exogenous chromophores, and subsequent US pressure wave generation for imaging. Photoacoustic (PA) imaging has been demonstrated to be useful for a variety of medical and biological diagnostic applications, including early cancer detection [11–14]. Biomarkers such as vascularity and hypoxia have been shown to have diagnostic value in the differential diagnosis of various types of cancers including breast cancer [15–17]. In addition, when PA is augmented with nano-sized contrast agents, it can provide a reliable platform for the molecular imaging of cancer and its sub-types [18–21]. In clinical applications, PA imaging has been shown to produce real-time molecular and functional information with high resolution at relevant depths [19,22].

**Figure 1.**
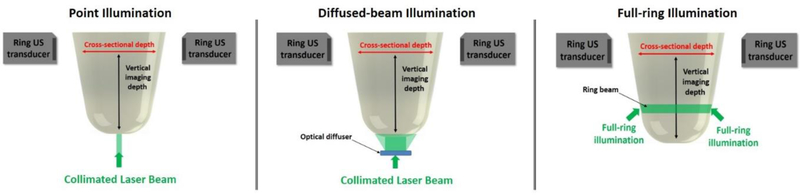
The three methods of illumination for photoacoustic tomography (PAT) imaging that are compared in this study, with the definitions of vertical and cross-sectional imaging depths.

Several PAT imaging systems with different illumination and acquisition modes have been developed for breast cancer imaging. However, the observed limitations of these systems are in part due to the non-optimum acoustic signal acquisition or illumination methodologies used. Our presented system is meant to be non-invasive (i.e., both illumination and acquisition are external), with the illumination and measurement system external to the imaged tissue. Our method images a cross-section inside the cylindrical US transducer array by illuminating the targeted area using a ring beam. Therefore, the light has to only diffuse half the tissue diameter that is encountered when using side illumination. Point or diffuse illuminations are suitable for imaging cross-sections close to the point of light entry [11,23–27], and the given fluence drops with light propagating through the tissue towards higher vertical depths as shown in Figure 1. This could make it difficult to access areas close to the chest well.

Other PAT imaging methods, such as the PA mammoscopy system [28], compress the tissue for better light penetration but can cause discomfort, or a loss of important PA biomarkers arises from the presence of blood by pushing the blood out of the tissue. One type of full-ring illumination system uses an acoustically penetrable optical reflector (APOR). However, APORs can only support low laser energies and US transmission through the reflector is highly angle-dependent [29–31]. Other illumination methods for deep tissue illumination include internal illuminations [32,33]. However, internal illumination is difficult to develop for breast imaging applications. Therefore, it is vital to develop an alternative solution for improving the uniformity of energy distribution within the breast tissue for more accurate PAT imaging.

Ultrasound tomography (UST) using a ring-shaped US transducer has shown promising results in breast cancer screening [34–38]. In this work, PAT imaging is combined with this novel full ring UST system. The PA imaging modality can be easily combined with UST since both modalities share the same acquisition hardware. For this reason, the addition of the PAT to the UST is straightforward and will provide valuable functional information about a given tissue and is expected to improve the diagnostic capability of breast US for physicians.

The design of our combined UST/PAT imaging system has been previously presented [39–42]. This setup uses a ring illumination in conjunction with a ring US transducer for combined UST/PAT imaging. The ring-shaped beam in this system is generated by using a cone mirror and a parabolic reflector. This work specifically compares three different illumination methodologies for PAT imaging: full-ring, diffuse, and point illuminations. Using new findings from the three methods, it aims to show that full-ring illumination is the most effective method for creating PAT images due to its inherent cross-sectional fluence uniformity across vertical imaging depths (Figure 1). This is especially important for breast cancer screening when imaging close to the chest wall proves difficult.

The three illumination methods are compared by imaging a three-layer polyvinyl chloride (PVC) tissue-mimicking phantom to gauge the advantages and disadvantages of common PAT imaging techniques. The experiments presented in this paper also all use the same data acquisition system and settings. Comparisons are made between PAT amplitudes for each cross-section and illumination methodology. Furthermore, the optimum position of the ring beam with respect to the targeted cross-section is examined.

## 2. Material and Methods

### 2.1. UST/PAT Acquisition System

**Figure 2.**
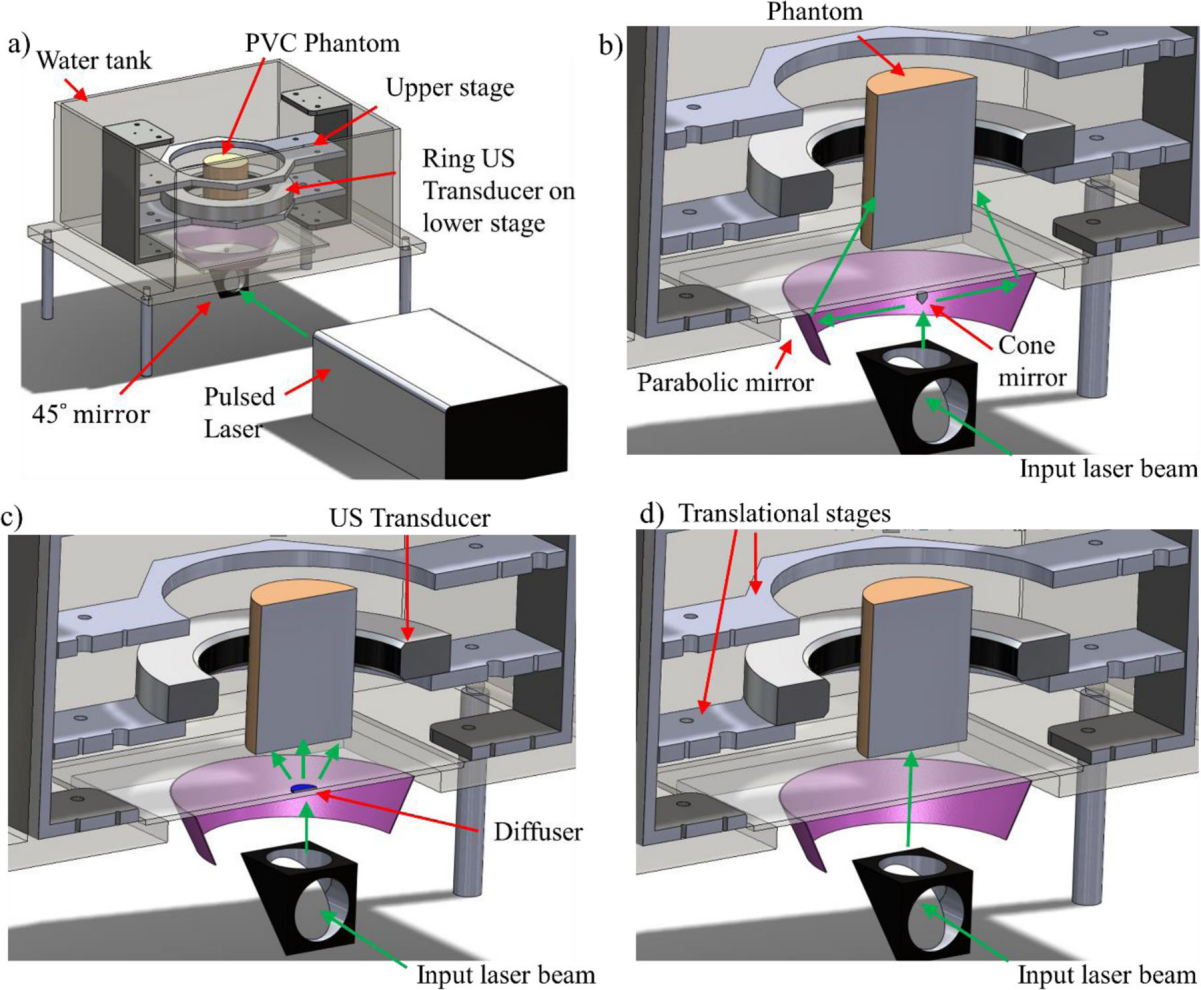
**(a)** PAT experimental setup showing the water tank, ring ultrasound (US) transducer, and the translational stages. The experimental setups for the **(b)** full ring, (c) diffuse-beam, and **(d)** point illumination of the phantom.

A 200 mm diameter, 256-element ring US transducer (Analogic Corporation, Canada) with a center frequency of 2 MHz and bandwidth of 60% was used for all data acquisition. The presented system has a measured lateral resolution of 1 mm as determined by measuring a 200 micrometer light-absorbing string. This transducer has an element pitch of 2.45 mm and a height of 9 mm. The scattered US waves from a PA imaging event are recorded by all 256 elements using a sampling frequency of 8.33 MHz. As shown in Figure 2a, the US ring transducer is housed in an acrylic tank and is supplied with degassed, distilled water. During PAT imaging, the ring US transducer uses a 10 dB linear, time gain compensation (TGC) for acquiring the data, which is designed to optimize the signal-to-noise ratio (SNR) for the given phantom.

### 2.2. Laser Source and Light Illumination Schemes

A tunable, 10 nanoseconds pulsed laser (Phocus Core, Optotek, Carlsbad, CA, USA) was used for all PAT imaging experiments. This laser generates around 100 mj per pulse at 532 nm. In the full-ring illumination mode, a large parabolic reflector (P19–0300, Optiforms Inc., Temecula, CA, USA) was used with a 10 mm diameter cone mirror (68–791, Edmund Optics, Barrington, NJ, USA) to create the 4 mm ring-shaped beam on the phantom surface (Figure 2a). Since the beam position is stationary, neither the cone mirror nor the parabolic reflector is mobile. The ring location was adjusted across each cross-section by translating the phantom in the vertical direction (Figure 2b). For the diffused-beam experiments, a 120 grit ground glass diffuser (DG10–120, Thorlabs Inc., Newton, NJ, USA) was placed in the laser light path inside the water tank after removing the cone mirror (Figure 2c). Finally, point illumination only uses the 45-degree mirror for directing the laser beam onto the phantom (Figure 2d).

### 2.3. Tissue-Mimicking Phantoms

**Figure 3.**
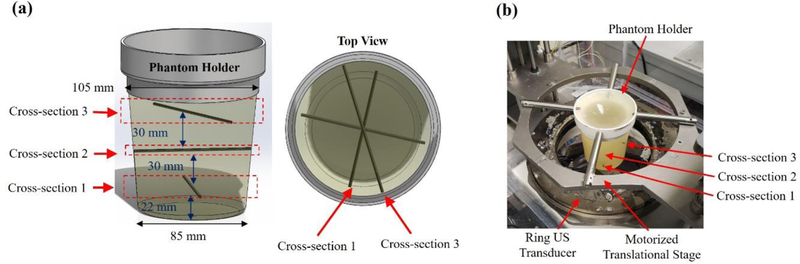
**(a)** Graph illustrates the polyvinyl chloride (PVC) phantom and graphite inclusions and their dimensions, **(b)** A photograph of the experimental setup including the ring US transducer. A motorized translational stage was used to adjust the position of the phantom to acquired images at multiple cross-sections.

The performance of the three different illumination methods was evaluated with regards to the PA imaging depth using a phantom made of polyvinyl chloride (PVC) (M-F Manufacturing Super Soft, Fort Worth, TX, USA), with 0.2% fine ground silica (US Silica MIN-U-SIL5, Stow, OH, USA) used as an optical diffuser. To create a PVC phantom [43], the plastisol was first mixed with the ground silica and then heated in a microwave to 170 °C. In a mold, three graphite rods with a 2 mm diameter were placed horizontally, and the PVC was poured to create the three layers as shown in Figure 3. After cooling, the phantom was removed from the mold and used for the described experiments. Graphite was chosen as an absorber due to its broadband absorption characteristics and ease of placement inside the phantom.

### 2.4. UST and PAT Image Reconstruction

In all instance, the waveform method was used to reconstruct the UST images [44], while filtered back-projection was utilized to reconstruct the PAT images [45]. For PAT back-projection reconstruction, the RF (radio frequency) values for each cross-section were averaged 10 times to increase the overall SNR. In UST mode, a 20 dB linear gain TGC was used for acquiring the US images, while in PAT mode, as previously described, a linear 10 dB TGC was used for data acquisition. For US imaging, the used TGC was optimized to minimize the transducer saturation, which resulted in cleaner images. This was done empirically. For PAT imaging, the value chosen was designed to reduce the signal emanating from other cross-sections from appearing in the cross-section of interest.

## 3. Results and Discussion

### 3.1. A Comparison of the Three Different Illumination Methods

**Figure 4.**
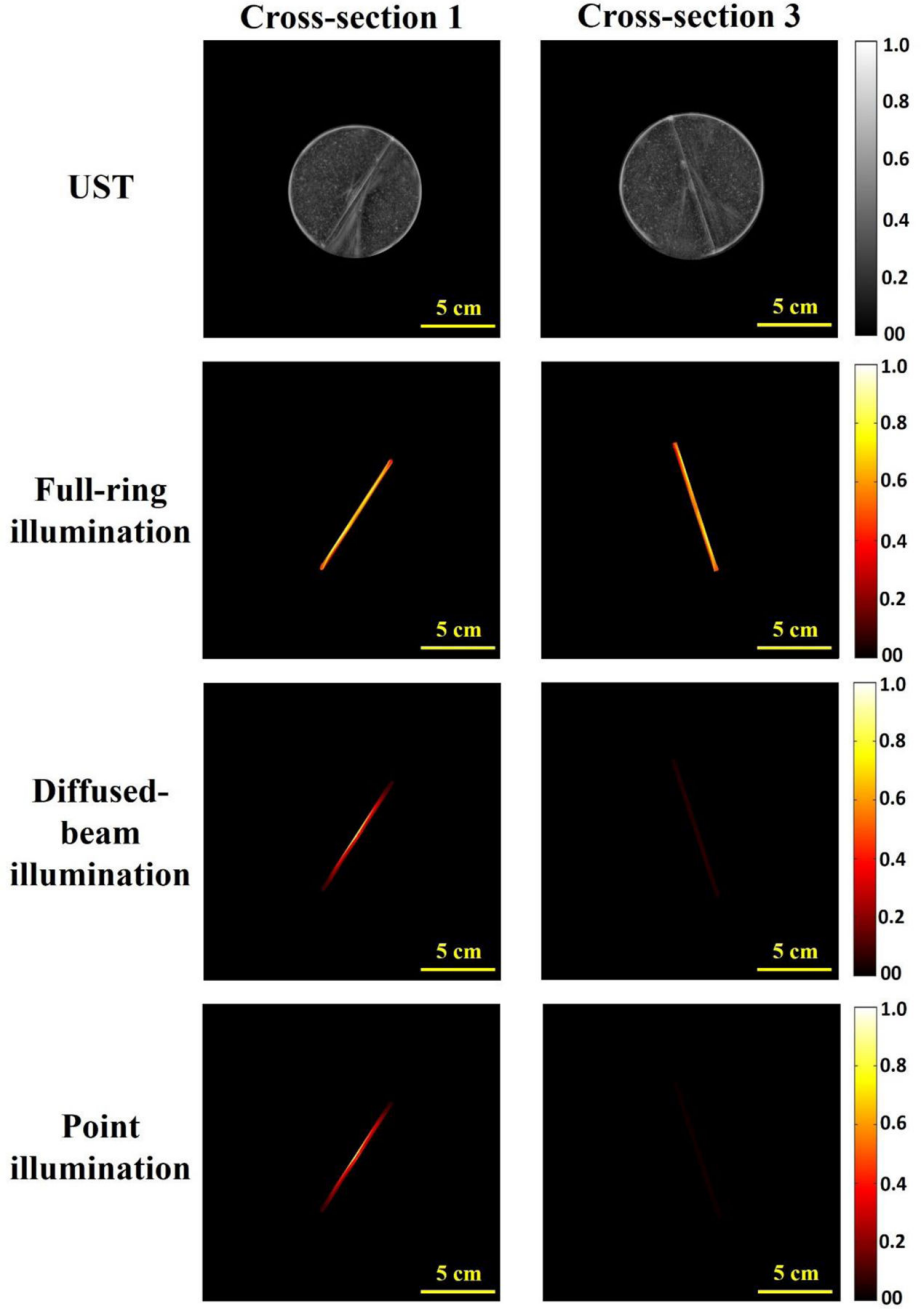
Ultrasound tomography (UST) and normalized PAT images of the PVC phantom with the graphite absorber using the three different illumination techniques for cross-sections 1 and 3.

The results discussed in this section focus on analyzing the PAT images and PA signal amplitudes from progressively deeper phantom cross-sections using the discussed illumination methodologies. Figure 4 shows the UST and PAT images for the first and third cross-sections, which are separated by 60 mm. The PAT images are masked based on the region of interest (ROI) as determined by the UST image. For each illumination method, the PA amplitude is normalized to the highest value for the method across all cross-sections. For example, for the full-ring illumination method, images were normalized to the highest amplitude of PA detected within the three slices, which are separated by 30 mm each. This allows for visualization of the effect of depth on the PA signal amplitudes for each illumination method. As can be seen in the PAT images, the graphite absorber is visible in the first and third layers (i.e., larger vertical depth) of the full-ring illumination method, which is not the case for the diffuse and point-source methods.

**Figure 5.**
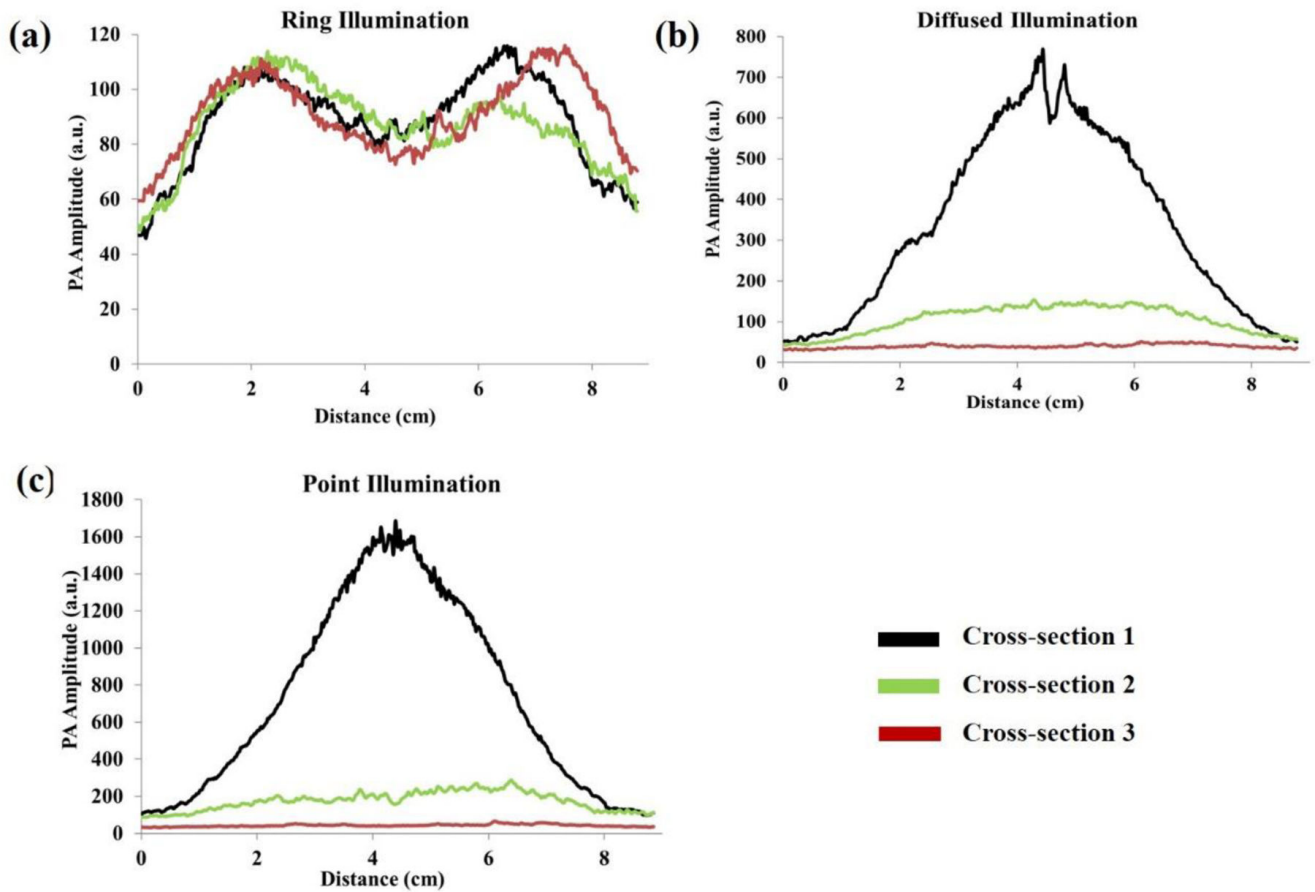
PA amplitude across the graphite absorber for three different cross-sections for: **(a)** full-ring, **(b)** diffused-beam, and **(c)** point illumination.

To further quantify the results shown in Figure 4, the PA signal amplitude across the graphite absorber for each illumination method, and for each cross-section, is plotted in Figure 5. For the full-ring illumination method, the PA values are nearly constant across the three cross-sections (Figure 5a). However, the peak amplitude value of the PA signal decreases by 25 times for point-source illumination and 15 times for the diffused-beam illumination between the first and third cross-sections. As seen by the uniformity in the amplitudes between three cross-sections for the full-ring illumination method, this imaging technique can provide a consistent image regardless of vertical depth. On the other hand, the diffused and point illumination methodologies show variance in PA amplitude signals. This finding demonstrates that the full-ring illumination is capable of providing sufficient fluences at lower vertical depths, which results in detectable and reliable PAT images across cross-sections.

**Figure 6.**
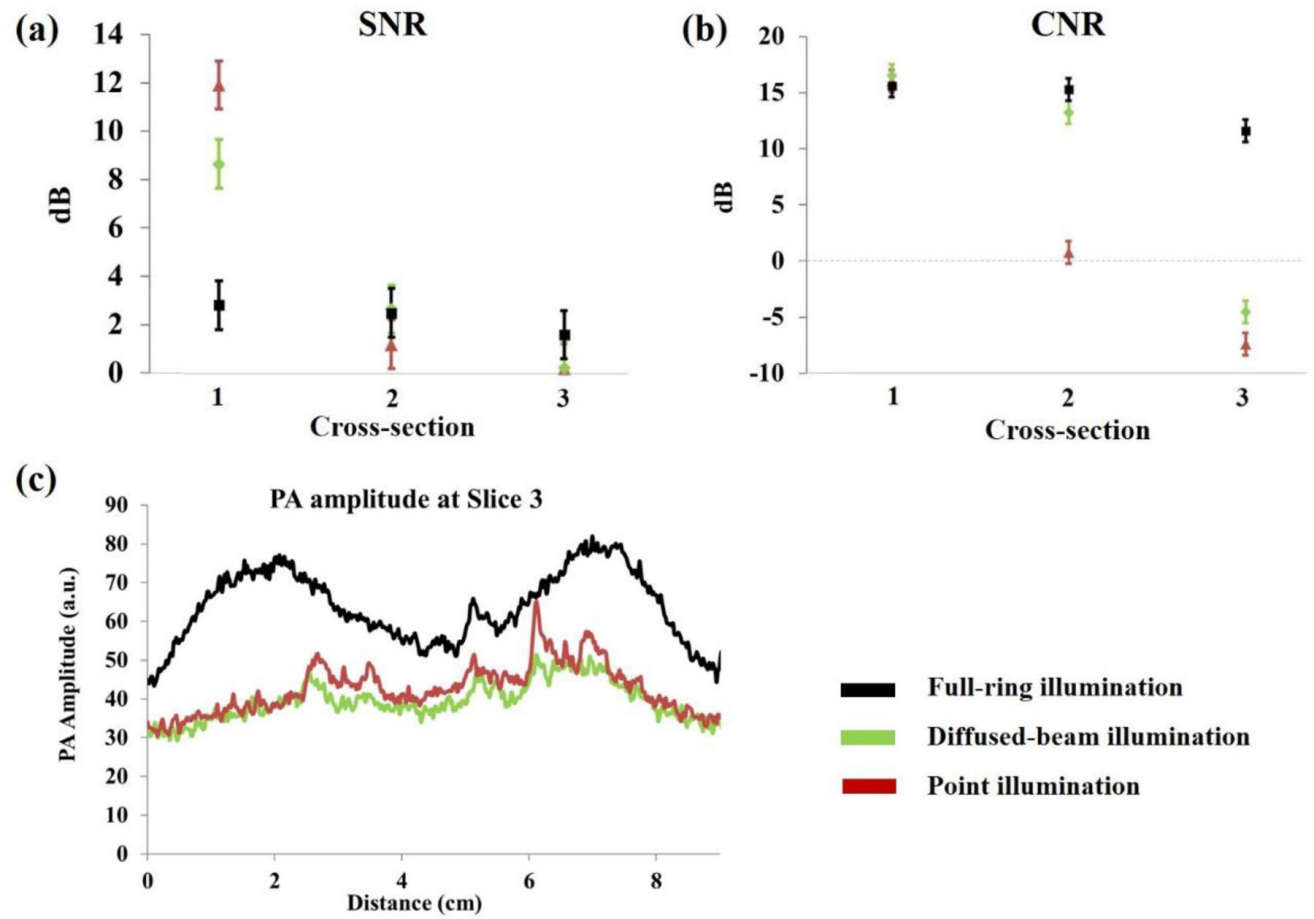
The signal-to-noise ratio (SNR) and contrast-to-noise ratio (CNR) of the PA amplitudes at three different cross-sections are plotted in **(a,b)**, respectively. For the full-ring illumination, the values were determined based on the illumination at 15 mm below the cross-section of interest, **(c)** Plots the PA amplitude for the top cross-section (cross-section 3) for full-ring, diffuse, and point illumination.

To further compare the performance of the three illumination strategies, the SNR and contrast-to-noise ratio (CNR) of the PAT images were measured. In the SNR, the value is calculated by:

$$\text{SNR}=20\times \text{log}10\left(\frac{M_{S}}{M_{B}}\right)$$

where *M*s refers to the mean of the PA signal, as marked by the US image region of interest, while *M*_*B*_ refers to the mean of the phantom background. The CNR is determined by:

$$\text{CNR}=20\times \text{log}10\left(\frac{M_{S}-M_{B}}{\sigma_{B}}\right)$$

where *M*s refers to the mean of the PA signal, Mg refers to the mean of the phantom background, and *σ*_*B*_ is the standard deviation of the phantom background. For the full-ring illumination method, the used PA amplitude values are from irradiating the target cross-section 15 mm below the cross-section of interest. As can be seen in Figure 6a,b, the SNR and CNR are nearly constant for the full-ring illumination across the three cross-sections, which is not the case for the diffuse and point illuminations. Figure 6c also plots the amplitudes across the graphite object at the third cross-section for all illumination methods. Based on the laser beam diameter of 8 mm; optical losses in the system; and 100 mJ input energy, the diffuse illumination method has a fluence of 9.3 mJ/cm^2^, compared to 175 mJ/cm^2^ for point illumination, and 7 mJ/cm^2^ for the full ring illumination. Full-ring calculations use a beam height of 4 mm circumferentially on a 9 cm diameter phantom, and diffused beam calculations use a beam diameter of 30 mm at the phantom. Even though point illumination has about 25 times the fluence of the full-ring illumination method, its amplitude is much smaller at this cross-sectional depth.

When compared to the diffused-beam and point illuminations, full-ring illumination has a higher PA amplitude at Cross-section 3. It has a near constant SNR, CNR, and PA signal amplitude across all cross-sections, making it an effective illumination method for breast imaging. Given that imaging breast regions close to the chest wall (i.e., large vertical depth) are clinically important, the full-ring illumination shows promise in accessing these regions and thus provides a means for reliable whole breast PAT imaging with a ring US transducer.

### 3.2. The PA Amplitude of the Targeted Cross-Section as a Function of Illumination Position

**Figure 7.**
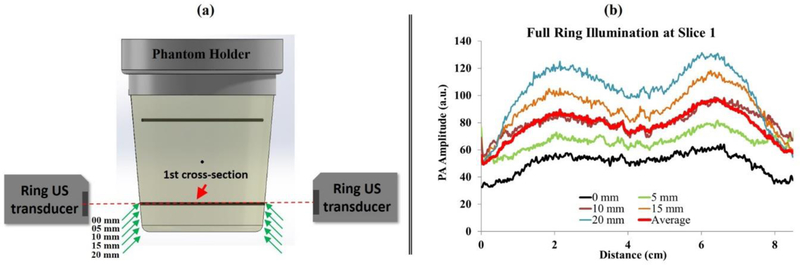
**(a)** The image shows the different positions of the ring beam based on the targeted cross-section (Cross-section 1). The targeted cross-section is located in the central field of view of the US elements. **(b)** PA amplitude at Cross-section 1 plotted as a function of illumination depth below the cross-section.

The optimum position for the full-ring beam was investigated by evaluating the effect of the distance between the ring-shaped beam and the targeted cross-section. The distance between the targeted cross-section and the full-ring beam was changed within a range of 0–20 mm. Zero millimeters represents the case where the ring beam is illuminating the targeted cross-section at the graphite rod, while the 20 mm case is when the ring beam is 20 mm below the graphite rod (Figure 7a). In this study, a selected cross-section was imaged while changing the illumination location from 0 to 20 mm. The five different positions chosen to illuminate the targeted cross-section of the graphite rod were 0,5,10,15, and 20 mm. A 532 nm laser with 100 mJ per pulse energy was used for this experiment. The location of the full-ring illumination was adjusted by translating the phantom in steps of 5 mm in the vertical direction.

In Figure 7b, the PA amplitude was measured by drawing a line across the targeted graphite for all five positions. The overall shape of the PA amplitude is constant while the maximum increases as one moves further below the desired imaging cross-section. This increased visibility could be due to the fact that the incidence angle of the beam illuminates the central part of the object more directly as it moves below the cross-section. A possible reason for the stronger PA signal, when illuminating 20 mm below the targeted cross-section, could the incident angle of the light diffusion within the tissue. In the current prototype, the ring mirror is illuminating the object cross-section at an angle of 39 degrees with respect to the object surface. The optimum illumination will occur if the ring beam falls normal to the surface. The 0 and 5 mm cross-sections use the US image as a mask to crop out the large PA peaks generated at the surface of the phantom. The graphite absorber was embedded within the PVC background with a margin from the surface. Here, we only evaluated the signal arising from the absorber. In cases where the illumination was coincident with the center of the transducer, a strong PA signal from the surface was observed (not shown in these masked images). Hence, imaging below the transducer can help to better visualize more central parts of the object due to elimination of a large PA signal arising from the light-entering surface.

The large PA signal at the surface is primarily due to the large fluence at the surface, which can affect the visualization of deeper regions due to a limited dynamic range in PA acquisition. This is not the case where the illumination was adjusted to 10 to 20 mm with respect to the center of the US detectors (i.e., illuminating below the imaged plane). The averaged PA amplitude from all five illumination methods (shown in red in Figure 7b) is similar to the 10 mm illumination results. It is worth mentioning that these results are not necessarily general for all ring illumination systems and are dependent on the incident angle of the beam with respect to the object (39 degrees in our case). The results might vary in other ring illumination systems if the incident angle is changed.

These findings are important because they help to define the illumination scenario based on the characteristics and size of the given object. For example, for a large diameter phantom one might need to acquire PAT images using more than one position of full-ring illumination to cover the regions closer to the outer surface and deeper regions (with illumination offset from the imaging plane). In addition, illuminating below the targeted cross-section (below the central line of the US elements) could help to limit the high PA signals that are generated from the outer surface of the scanned object. The used illumination method has a significant effect on the imaging’s vertical depth, which is significant when visualizing anatomy such as the breast.

## 4. Conclusions

Phantoms with horizontal graphite absorbers were used to examine the efficacy of full-ring, diffuse, and point illumination PAT imaging using a ring US transducer. The full-ring illumination was able to provide a more uniform fluence irrespective of the vertical depth of the imaged cross-section, while the point source and diffused-beam resulted in a high fluence at the point of entry, which diminished with vertical depth. In addition, the preliminary results indicate that illuminating an object 10–15 mm below the imaged cross-section of interest might be optimal for this system since it avoids large surface reflections and provides better coverage to the cross-section.
